# Enhanced Anti-Tumor Efficacy of Paclitaxel Nanoparticles via Supramolecular Self-Assembly with Pterostilbene

**DOI:** 10.3390/ph18121828

**Published:** 2025-12-01

**Authors:** Xin Liang, Ru-Yan Wen, Jie-Feng Chen, Hai-Li Wu, Ling Chen, Ning Lin, Xue-Mei Liu, Qing Chen

**Affiliations:** 1 Institute of Traditional Chinese and Zhuang-Yao Ethnic Medicine, College of Pharmacy, Guangxi University of Chinese Medicine, Nanning 530200, China; liangjinyuan77@petalmail.com (X.L.); wenruyan@126.com (R.-Y.W.); 17878920732@163.com (J.-F.C.); wuhaili2023@stu.gxtcmu.edu.cn (H.-L.W.); 17816193372@163.com (L.C.); linning@gxtcmu.edu.cn (N.L.); 2 Guangxi Innovation Center of Zhuang Yao Medicine, Nanning 530200, China

**Keywords:** paclitaxel, pterostilbene, co-amorphous, anti-tumor effects, synergism activity

## Abstract

**Background**: Paclitaxel (PTX), a taxane chemotherapy drug, is widely regarded as one of the most potent and clinically effective treatments for advanced and resistant cancers. However, paclitaxel’s poor bioavailability is attributed to its unfavorable physicochemical properties, including low solubility and permeability. Nanosizing and multidrug combination strategies have emerged as key approaches to enhance the formulation of such compounds. Pterostilbene (PTE), a polyphenolic compound, possesses extensive anti-cancer properties and favorable hydrogen bond formation sites. In this study, PTE was employed to co-assemble with PTX to improve its physicochemical properties and enhance therapeutic efficacy. **Methods**: Paclitaxel-pterostilbene nanoparticles (PTX-PTE NPs) were characterized by differential scanning calorimetry (DSC), powder X-ray diffraction (PXRD), Fourier transform infrared spectroscopy (FT-IR) and scanning electron microscopy (SEM). **Results**: PTX-PTE nanoparticles significantly improved the water solubility (7fold increase) and cytotoxicity of paclitaxel in tumor cells. The enhanced antitumor efficacy was achieved through P-gp and CDK1 protein downregulation, increased drug accumulation, and cell cycle inhibition. **Conclusions**: These improvements are attributed to the nanoparticles’ amorphous structure and nanoscale properties. In addition, the combined use of PTX and PTE significantly enhanced the cytotoxicity against human non-small cell lung cancer A549 cells. PTX-PTE nanoparticles show promise for improving drug delivery and overcoming multidrug resistance in A549 cells.

## 1. Introduction

Paclitaxel (PTX), as a first line therapeutic agent, is widely used in the treatment of various malignant tumors [1]. PTX is a precious natural product derived from Taxus chinensis, with outstanding anti-cancer properties. It induces cytotoxicity in cancer cells by penetrating the nano-metersized pores in the microtubule wall and interacting with tubulin on the lumen surface, thereby disrupting the dynamics of microtubules. PTX’s low toxicity, high efficiency, and broad-spectrum application have led to its approval for the treatment of various diseases. The wide success and expanding applications of PTX have resulted in an increased market demand [2]. However, its limited water solubility poses a significant challenge to its pharmaceutical efficacy. Its water solubility is at the limit of 0.3–0.5 μg/mL [3]. In recent years, research efforts have predominantly focused on liquid-based delivery systems, including prodrugs, liposomes, and micelles, to enhance PTX solubility [4]. While these systems have partially addressed the issue of poor solubility, they continue to encounter numerous practical challenges. For instance, the relatively low drug loading and the physical and chemical property improvement of a single drug are difficult to deal with increasingly drug-resistant tumor cells [5,6,7,8]. Consequently, there is an urgent need for the development of safer and more effective drug delivery systems capable of improving PTX performance in both in vitro and in vivo contexts. A recent study has experimentally validated the synergistic therapeutic effect of quercetin and PTX against ovarian cancer. Compared to monotherapy, the combinatorial treatment markedly enhanced apoptotic activity in both SKOV3 and A2780 cell line models. Mechanistic profiling further demonstrated that this synergy arises from transcriptional downregulation of oncogenic drivers ERBB2 and BIRC5, coupled with CASP3-dependent activation of apoptosis-related signaling pathways [9]. Furthermore, clinical applications of PTX are often hindered by tumor cell drug resistance [10]. To counteract this resistance, combination therapy strategies involving other drugs have been widely adopted. Among them, natural small molecule compounds have received extensive attention due to their low toxicity and excellent biocompatibility, such as flavonoids and polyphenols. The construction of a single amorphous phase system incorporating the active pharmaceutical ingredient and natural small molecules has demonstrated the potential to markedly enhance drug solubility and dissolution rate while leveraging the synergistic effects of multi-drug combinations [11,12,13]. Therefore, employing small molecules as co-amorphous therapeutic agents represents a promising approach to improve the solubility and therapeutic efficacy of PTX.

Pterostilbene (PTE), a non-flavonoid polyphenolic compound, exhibits a broad spectrum of pharmacological activities, including anti-inflammatory, antioxidant, and anti-cancer properties [14]. In a previous study it was shown that different degrees of susceptibility were observed in various cancer cells. A375 melanoma and A549 lung cancer cells with lower Heat Shock Protein 70 (HSP70) levels were more sensitive to PTE, while HT29 colon cancer and MCF7 breast cancer cells with higher HSP70 levels were more resistant to it. The data indicated that lysosomal membrane permeabilization was the main cell death pathway triggered by PTE [15]. In cancer treatment, PTE functions as a potential sensitizer, augmenting the effectiveness of various tumor therapies [16,17]. It has shown promise in the prevention and treatment of diverse cancers, including breast, colon, lung, cancers [18]. Its mechanisms of action encompass the regulation of cell cycle dynamics, induction of programmed cell death, inhibition of angiogenesis, and suppression of cancer cell metastasis [18,19].

Nanotechnology has extensive applications in the medical field, particularly demon strating significant advantages in targeted delivery of anti-cancer drugs [20]. Nanomedicine drug delivery systems can effectively improve the apparent state of the original drugs, such as solubility, stability and overall therapeutic effect, thereby overcoming the physiological barriers of drug accumulation at tumor sites, reducing off-target toxicity, prolonging the bioavailability of drugs, and ultimately enhancing the therapeutic effect [21]. Notably, nanomedicine delivery systems can be constructed without relying on inactive dedicated carriers, a strategy known as carrier-free drug delivery systems [22]. However, most nano-drugs still require surfactants or amphiphilic structures to maintain their stability and functionality, which demands that such stabilizers have high safety [23], such as Polyvinylpyrrolidone K30 (PVP-K30). PVP-K30 is a hydrophilic and non-toxic polymer with excellent biocompatibility, which can enhance the stability of nano-systems. It has been applied in various nano-preparations [24,25]. In recent years, carrier-free drug delivery systems have been widely applied in cancer treatment due to their high drug loading capacity, low off-target toxicity, and minimal immunogenicity [26]. Additionally, self-assembly technology has shown great potential in the development of new drugs. In self-assembled nanomedicine delivery systems, drug small molecules spontaneously form nanoscale carrier complexes through non-covalent bonds, including van der Waals forces, hydrogen bonds, π-π stacking, halogen bonds, hydrophobic interactions, and ionic bonds [27]. Amorphous solid dispersions, due to their high energy state, are prone to recrystallization during storage, which can affect the stability of the amorphous form. However, due to the strong interactions between co-amorphous drugs, they also exhibit strong interactions at the physical state or solid-state level, which can effectively prevent drug recrystallization [28,29]. These complexes can not only combine with photosensitizers or other drug molecules to form multifunctional nanomedicines targeting tumor tissues but also reveal the specific interaction mechanisms between drug components, providing theoretical basis and practical clues for more efficient combination therapy.

For tumor neovascularization, the cut-off size of capillaries is approximately 300–500 nm, which means that particles larger than this size cannot seep out of the blood, while smaller particles are more likely to penetrate into the tumor [30]. Prior research investigating 10 nm and 100 nm DOX-LG formulations revealed enhanced cellular internalization efficiency in the 10-nanometer system. Subsequent in vitro analyses further confirmed superior cytotoxic effects exhibited by the nanoscale DOX-LG platform measuring 10 nm in diameter [31]. Exploring feasible oral nano-delivery systems represents a promising direction for improving the clinical application prospects of PTX. The particle size of the drug has a significant impact on gastrointestinal absorption. To effectively deliver nanoparticles to the intestinal epithelium or systemic circulation, nanoparticles must first deposit and then penetrate the mucosal layer to avoid intestinal clearance. Larger nanoparticles (>600–1000 nm) mostly remain in the lumen and are rapidly cleared by the intestine. Medium-sized nanoparticles (>300 nm) deposit in the mucosal layer and are trapped by its hydrogel network. Smaller nanoparticles (<200 nm) can effectively penetrate the mucus layer to reach the intestinal epithelium, and even smaller nanoparticles are preferentially internalized by intestinal enterocytes and M cells and are more effectively transported to the systemic circulation than larger NPs [32].

Therefore, this paper aims to achieve better therapeutic effects by reducing the drug particle size through the screening of preparation conditions. This study is the first to explore the construction of PTX and PTE in a non-covalent form through anti-solvent precipitation to develop paclitaxel-pterostilbene nanoparticles (PTX-PTE NPs). The method was developed to enhance solubility and maximize the anti-A549 lung cancer cell effects of the two compounds. The preparation process was optimized using single-factor and orthogonal experiments, focusing on particle size and polydispersity index (PDI). In vitro tests verified improved solubility and anti-A549 lung cancer cell efficacy of PTX-PTE NPs. Overall, this study proposes a highly innovative drug delivery strategy with the potential to significantly enhance the solubility and therapeutic efficacy of poorly soluble drugs.

## 2. Results

### 2.1. Optimization of Anti-SOLVENT Precipitation

#### 2.1.1. Screening Study on the Molar Ratio of PTX to PTE

The effect of the molar ratio of PTX to PTE on the particle size of PTX-PTE NPs is pre sented in Table 1. The molar ratio of PTX to PTE significantly influences both the particle size and polydispersity index (PDI) of the resulting nanoparticles. When the PTX: PTE ratio was set at 2:1, the nanoparticles exhibited the largest average size (179.50 ± 2.0 nm), likely due to insufficient PTE content, which may lead to drug molecule aggregation. As the ratio was adjusted from 1:1 to 1:3, a gradual decrease in particle size was observed, ranging from 163.00 nm to 145.87 nm. However, at the 1:4 feeding ratio, the particle size of the nanoparticles increased to 174.87 ± 1.1 nm. These findings highlight the critical role of the PTX:PTE ratio in determining nanoparticle size and PDI. The PDI of PTX-PTE NPs prepared at different feeding ratios (from 2:1 to 1:4) showed significant variations. It is speculated that the differing ratios may have affected the binding efficiency and assembly stability between PTX and PTE, leading to the formation of nanostructures with incomplete binding or heterogeneous morphology, thereby resulting in a broadened particle size distribution [33]. However, the PDI of NPs prepared at all ratios remained below 0.3 [34,35], meeting the fundamental requirement for monodispersity in nanopharmaceutical formulations. Taking all factors into consideration, the formulation with a feeding ratio of 1:3, which exhibits a particle size of 145.87 ± 1.4 nm, was selected as the optimal choice due to its relatively low PDI and more desirable nanoparticle size.

#### 2.1.2. Screening of the Dosage of PVP-K30

The influence of the dosage of PVP-K30 on the particle size of PTX-PTE NPs is shown in Table 2. PVP-K30 is a widely utilized stabilizer for drug carriers, and its dosage exerts a critical influence on the physicochemical properties of nanoformulations [36]. The group with a PVP-K30 concentration of 1.5 mg/mL exhibited the smallest particle size among all experimental groups and fulfilled the criteria for nanoformulations (PDI < 0.3). In contrast, other groups displayed varying degrees of increased particle sizes and higher PDI values. Surfactant monomers adsorb onto the nanoparticle surface via their hydrophobic tails, while their hydrophilic heads interact with the polar solvent, effectively reducing interfacial tension and system free energy, thereby enhancing nanoparticle stability; if the concentration is insufficient to achieve complete surface coverage, particle aggregation and an increase in PDI will occur [37]. Based on these findings and the established requirements for nanoscale drug delivery systems, a PVP-K30 dosage of 1.5 mg/mL was determined to be optimal.

#### 2.1.3. Screening of the Ratio of Organic Phase to Aqueous Phase

The analysis of nanoparticle size and PDI data under varying organic-to-aqueous phase ratios, as presented in Table 3, demonstrates that the organic phase-to-aqueous phase ratio exerts a significant influence on the physicochemical properties of the nanoformulation. Overall, as the organic phase ratio increases progressively from 1:6 to 1:12, the particle size exhibits a fluctuating pattern—initially increasing, followed by a decrease, and subsequently another increase. The smallest average particle size (143.1 ± 1.2 nm) is observed at an organic phase-to-aqueous phase ratio of 1:10, which outperforms other tested ratios. These findings suggest that a moderate increase in the proportion of organic phase may facilitate the homogeneous dispersion of drug molecules and promote the stable formation of crystal nuclei.

As presented in Table S1, the analysis of nanoparticle size and polydispersity index (PDI) across varying PTX feed amounts demonstrates that the PTX dosage significantly influences the particle size of the nanoformulation. Specifically, a progressive increase in particle size was observed as the PTX feed amount increased from 10 mg to 25 mg. According to the data in Table S1, the smallest average particle size of 109.6 ± 0.3 nm was achieved at a PTX feed amount of 10 mg/mL. However, in consideration of the required PTX dosage for subsequent drug activity assays, a feed amount of 15 mg/mL was selected, yielding an average particle size of 137.6 ± 3.0 nm and a PDI of 0.142 ± 0.014. Furthermore, regarding the influence of reaction temperature on the formation of PTX-PTE NPs, there is a notable observation as shown in Table S2. At 30 °C, nanoscale particles could not be formed, and instead, irregular blocky precipitates were observed. In contrast, uniform nanoscale particles were successfully obtained at temperatures ranging from 35 °C to 40 °C. Based on the Twelve Principles of Green Chemistry, which recommend conducting chemical reactions at ambient temperature and pressure whenever possible [38], a reaction temperature of 35 °C was ultimately adopted.

The optimal preparation conditions for PTX-PTE NPs are as described in “2.2. Preparation of PTX-PTE NPs”. Furthermore, the experimental results demonstrate a high degree of conformity with the optimal parameter levels obtained through orthogonal experimental design, as illustrated in Tables S3 and S4.

### 2.2. Characterization of PTX-PTE NPs

**Figure 1:**
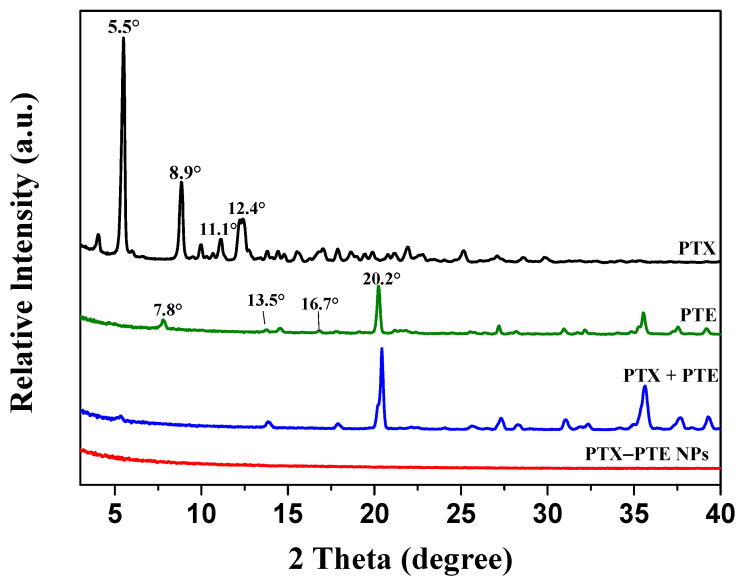
PXRD patterns of PTX, PTE, PTX + PTE and PTX-PTE NPs.

The formation of multi-drug co-crystals often leads to alterations in the physicochemical properties of drugs [39]. PXRD is widely recognized as an effective technique for assessing changes in drug crystallinity [40]. The PXRD patterns of PTX, PTE, PTX + PTE and PTX-PTE NPs are presented in Figure 1. PTX exhibits distinct diffraction peaks at 2θ angles of 5.5°, 8.9°, 11.1°, and 12.4°, consistent with previous studies [41]. Similarly, PTE displays characteristic diffraction peaks at 2*θ* of 7.8°, 13.5°, 16.7°, and 20.2° [42], PTX + PTE is manifested as the physical superimposition state of PTX + PTE, whereas the PTX-PTE NPs shows only a broad diffraction halo without discrete peaks, suggesting the formation of an amorphous state between PTX and PTE [43].

**Figure 2:**
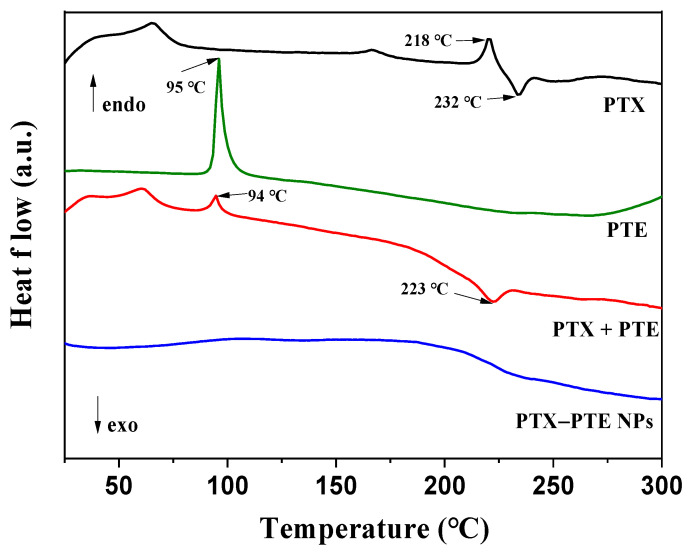
DSC curves of PTX, PTE, PTX + PTE and PTX-PTE NPs.

To confirm the amorphous nature of the PTX-PTE NPs, thermal analysis was employed to investigate its phase transition behavior. As shown in the DSC thermogram (Figure 2), PTX exhibits an endothermic peak at 218 °C followed by an exothermic peak at 232 °C [44], which aligns with its known melting point. PTE also demonstrates a sharp endothermic peak at 95.7 °C, consistent with literature reports [45]. PTX + PTE simultaneously possesses the thermal variations of both PTX and PTE. In contrast, the PTX-PTE group displays characteristics distinct from those of crystalline compounds. Its DSC curve reveals a single glass transition temperature typical of amorphous materials, with the disappearance of the original melting peaks of both PTX and PTE [46]. These findings, together with the PXRD results, support the presence of a co-amorphous system formed between PTX and PTE.

**Figure 3:**
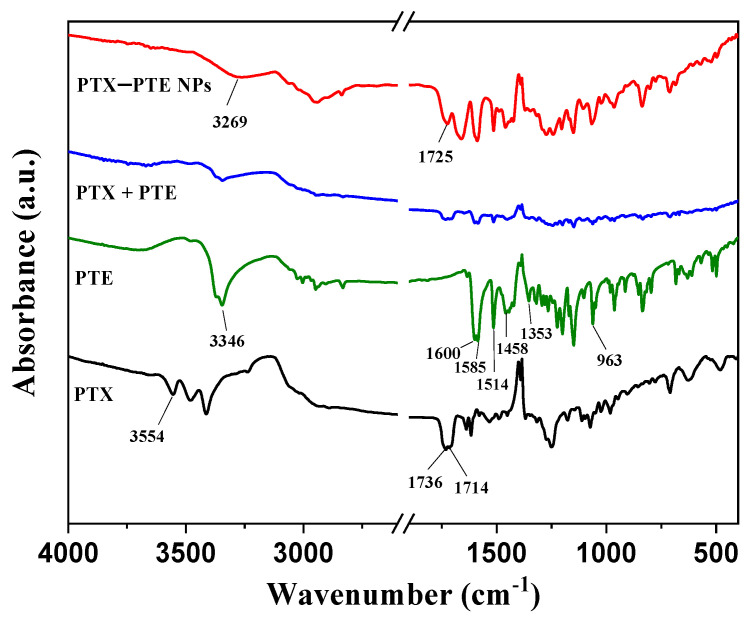
FT-IR spectra of PTX, PTE, PTX + PTE and PTX-PTE NPs.

Infrared spectroscopy (Figure 3) further elucidates the molecular interactions within the system. FTIR analysis of PTE revealed characteristic absorption bands at 1600.15, 1585.55, 1514.71, 1458.43, 1353.35, and 963.31 cm^−1^, corresponding to the stretching vibrations of aromatic -C=C-, alkenes -C-C-, aromatic -C=C-, aromatic -C=C-, -C-O- and trans-alkenes -C=C-, respectively, similar to those reported in reference [47]. PTX, on the other hand, showed characteristic absorption bands at 1736 and 1714 cm^−1^, corresponding to the stretching vibration of C=O, similar to those reported in reference [48]. The absorption bands at 3346 cm^−1^ in PTE and 3554 cm^−1^ in PTX are attributed to the stretching vibration of O-H. The FTIR spectrum of PTX + PTE indicates a physical mixture of PTX and PTE, suggesting no interaction between them. However, the FTIR spectrum of the PTX - PTE group shows a broad peak at 3269 cm^−1^, indicating a shift in the phenolic hydroxyl group of PTE from 3346 cm^−1^ to 3269 cm^−1^. Additionally, the vibration peak corresponding to the carbonyl group shifts to 1725 cm^−1^, suggesting that the development of the co-amorphous system might be due to the interaction between the carbonyl group of PTX and the phenolic hydroxyl group of PTE [48]. Based on the single-factor investigation of PTX-PTE NPs, especially the influence of temperature on the formation of PTX-PTE NPs, the co-precipitation of PTX and PTE is the key to the formation of PTX-PTE NPs. According to reference [49], PTX and PTE need to reach a thermodynamic equilibrium state during the co-precipitation process in order to form self-assembly. This is a different point from a simple physical mixture.

**Figure 4:**
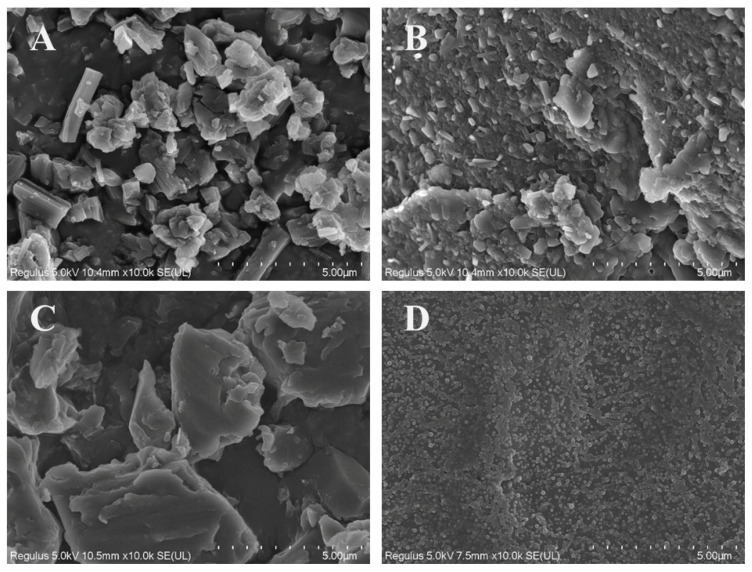
SEM images of PTX (**A**), PTE (**B**), PTX + PTE (**C**) and PTX-PTE NPs (**D**) magnified 10,000 times.

**Figure 5:**
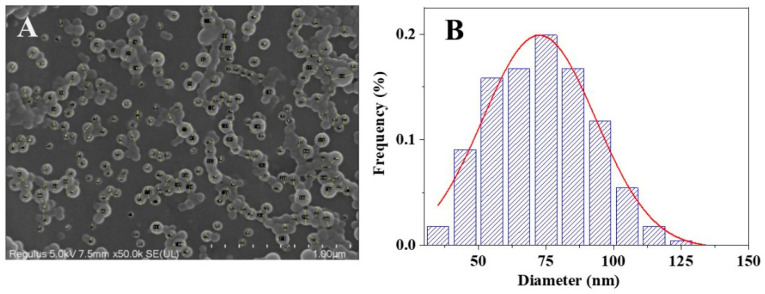
SEM image of PTX-PTE NPs at 50,000 times magnification (**A**) and in its particle size distribution graph, the red line represents the curve obtained through Gaussian fitting (**B**).

Figure 4 shows the SEM images of PTX, PTE, PTX + PTE and PTX-PTE NPs at the same magnification (×10,000), illustrating their microscopic morphological characteristics. These images provide direct morphological evidence for the physical properties of the nanoformulations. As shown in the SEM images, both PTX and PTE raw materials exhibit distinct crystal features. Specifically, PTX, PTE and PTX + PTE all show uneven particle size distribution, with clear edges and large and irregular particles as their characteristics. Figure 5 shows the SEM image and particle size distribution of PTX-PTE NPs at a magnification of 50,000 times. The particle size is 72.5 ± 0.9 nm, which is smaller than the particle size of 143.1 ± 1.2 nm detected by the Mastersizer 2000 (Malvern Panalytical, Malvern, UK) in the suspension state. Based on the SEM images of PTX-PTE NPs, it is inferred that SEM provides the number-weighted size of dried particles, whereas laser diffraction (Mastersizer 2000) measures the volume-weighted hydrodynamic diameter in a dispersed system. Due to the presence of a solvation layer and slight aggregation, the detected particle sizes tend to expand, leading to discrepancies between the measurements obtained via SEM and Mastersizer 2000. Compared with the raw material drug, PTX-PTE NPs shows significant morphological differences. The nanoparticles are mainly spherical or nearly spherical with a uniform size distribution, which is distinctly different from the crystal morphology of the raw materials. These observations suggest that during the self-assembly process, the morphological structure of PTX and PTE undergoes a fundamental transformation, changing from the original columnar or needle-like crystal form to uniform spherical nanoparticles.

### 2.3. In Vitro Dissolution Experiment

**Figure 6:**
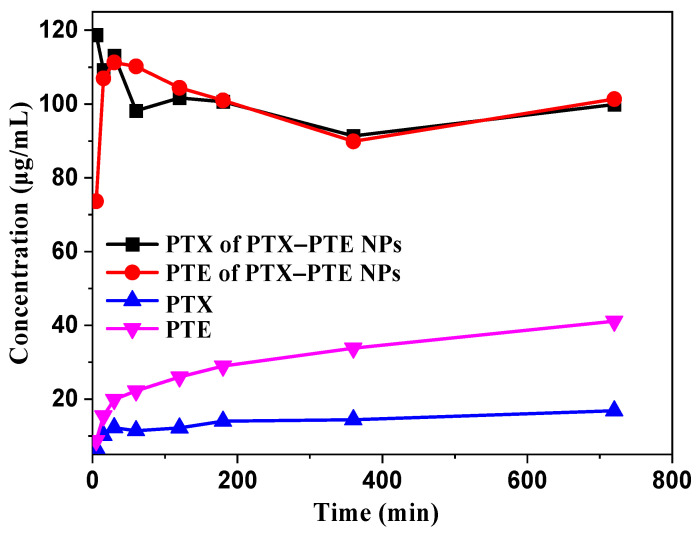
Dissolution profiles of PTX and PTE from crystalline PTX, PTX + PTE and PTX-PTE NPs.

As illustrated in Figure 6, the dissolution profiles of PTX-PTE NPs and the raw drugs were evaluated in a 0.3% SDS aqueous solution. Upon formation of the nanocomplexes, PTX-PTE NPs rapidly attained peak mass concentration in the dissolution medium, followed by a gradual decline, exhibiting a characteristic “spring-parachute” effect. This phenomenon can be primarily attributed to the higher free energy of amorphous drugs, whose original long-range ordered crystalline structures are disrupted. As a result, they can dissolve rapidly without the need to overcome lattice energy during the dissolution process [50]. However, upon exposure to the dissolution medium, the surface of the amorphous drug may undergo recrystallization, which could hinder further dissolution of the inner layers [51]. Due to its hydrophobic property, PTX maintains a relatively low solubility, with a maximum concentration of only 16.03 μg/mL in an aqueous solution containing 0.3% SDS. Analysis of the dissolution data reveals an overall dynamic trend indicating that PTX-PTE NPs demonstrate significantly enhanced dissolution performance compared to the raw PTX. Specifically, in the 0.3% SDS aqueous solution, the peak concentration of PTX in PTX-PTE NPs was approximately 7 times higher than that of the raw PTX drug, further confirming the beneficial effects of amorphization and nanoscale size on the drug dissolution behavior. This “synergistic effect” is manifested as a higher dissolution rate in the dissolution experiment and is further verified in the in vitro cell model for its biological performance advantages.

### 2.4. In Vitro Evaluation of Inhibitory Activity on Human Lung Cancer A549 and A549/T Cells

**Figure 7:**
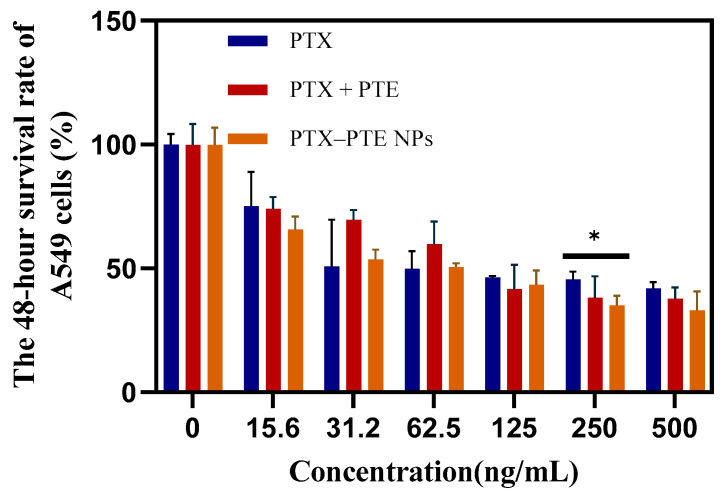
The effects of PTX + PTE, PTX-PTE NPs and PTX on A549 cells. The data represented as the mean ± SD (n = 3). * *p* < 0.05.

**Figure 8:**
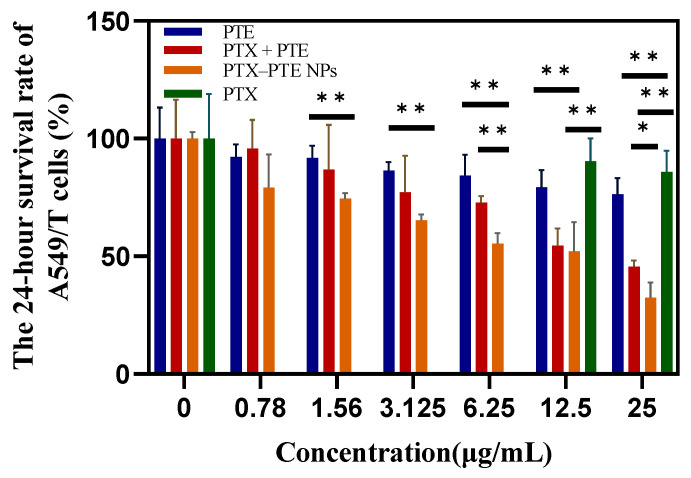
The effects of PTX + PTE, PTX-PTE NPs, PTX and PTE on A549/T cells after 24 h of administration. The data represented as the mean ± SD (n = 3). * *p* < 0.05, ** *p* < 0.01.

**Figure 9:**
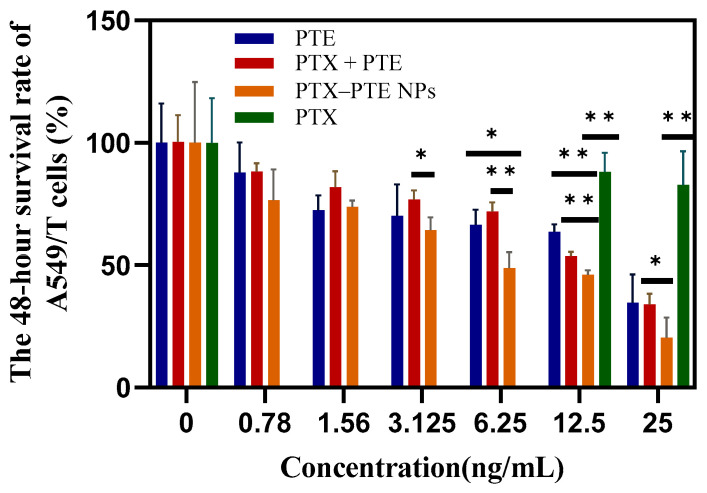
The effects of PTX + PTE, PTX-PTE NPs, PTX and PTE on A549/T cells after 48 h of administration. The data represented as the mean ± SD (n = 3). * *p* < 0.05, ** *p* < 0.01.

**Figure 10:**
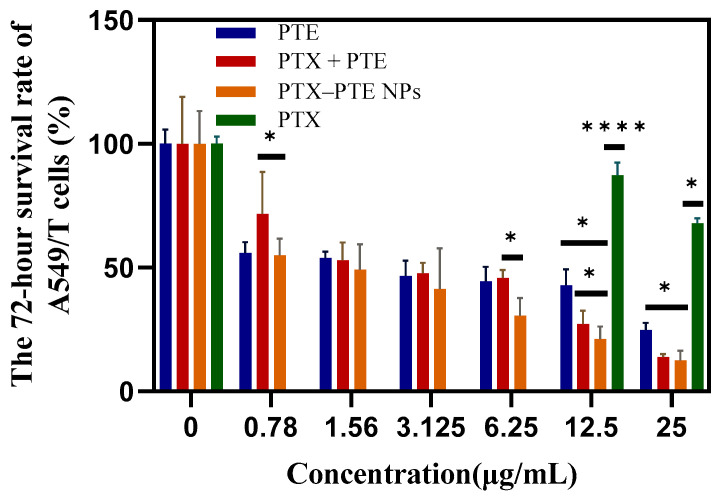
The effects of PTX + PTE, PTX-PTE NPs, PTX and PTE on A549/T cells after 72 h of administration. The data represented as the mean ± SD (n = 3). * *p* < 0.05, **** *p* < 0.0001.

As shown in Figure 7, PTX treatment led to a concentration-dependent decrease in A549 cell viability, indicating that PTX has a significant cytotoxic effect on A549 cells. After PTE treatment, comparable cell viability inhibition was observed in both A549 and A549/T cells, suggesting that PTE can maintain a relatively consistent inhibitory effect even in the presence of PTX-resistant A549/T cells (Figure S1). This indicates that the combined administration of PTE and PTX can achieve a synergistic inhibitory effect on A549/T cells. PTX-PTE NPs demonstrated enhanced cytotoxicity against A549/T cells at 24 h, 48 h and 72 h, with a more pronounced inhibitory effect compared to PTX + PTE (Figure 8, Figure 9 and Figure 10). Therefore, PTX-PTE NPs inhibit the activity of A549/T cells through the synergistic interaction between PTX and PTE.

### 2.5. The Effects of PTX, PTE and PTX-PTE NPs on the Expression Levels of Multidrug Resistance-Related Proteins in A549/T Cells

**Figure 11:**
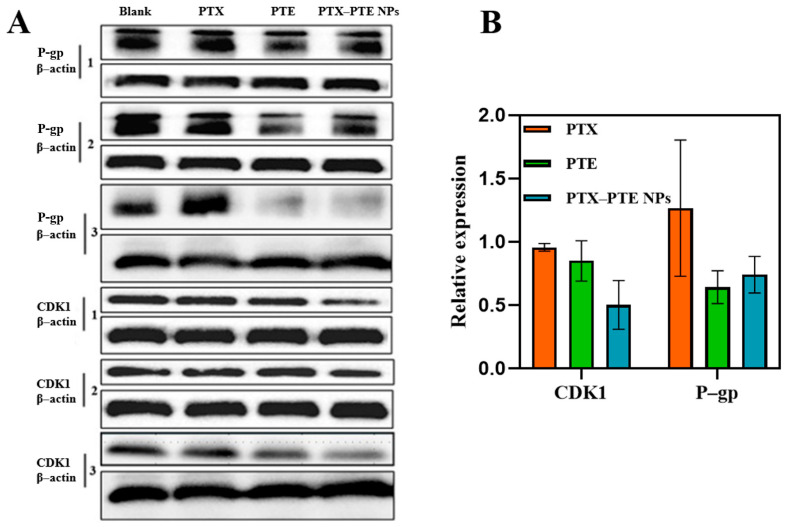
Effect of combined treatment with PTX and PTE on the expression of P-gp and CDK1 proteins in A549/T cells. (**A**) The protein expression levels in A549/T samples were detected by Western blotting. (**B**) The protein expression induced by combined treatment was quantified byoptical density analysis and normalized to β-actin as an internal reference.

As shown in Figure 11, in A549/T cells, both P-gp and CDK1 protein levels remained relatively high in the PTX group, while the protein levels in the PTE group were lower than those of P-gp and CDK1 in the PTX group. Combined with the inhibitory effects of PTX and PTE on A549/T cells, these findings suggest that A549/T cells may develop resistance to PTX through high expression of P-gp and CDK1. Compared with monotherapy, PTX-PTE NPs further reduced the protein expression levels of both CDK1 and P-gp. This is consistent with the significantly enhanced inhibitory effect of PTX-PTE NPs on A549/T cells compared to the raw drug groups. These results demonstrate that PTX-PTE NPs disrupt the normal cell cycle process and inhibit cell viability by down-regulating the expression of P-gp and CDK1 proteins.

## 3. Discussion

This study successfully developed PTX-PTE NPs with an average particle size of approximately 150 nm and a polydispersity index (PDI) below 0.3 using a single-factor experimental approach. The resulting complex demonstrated excellent uniformity in particle size distribution. During the preparation process, several critical parameters, such as solvent ratio and the feed ratio of active components were systematically optimized. Precise regulation of these parameters was essential for ensuring the stability and reproducibility of the formulation. Through continuous refinement of the preparation methodology, both miniaturization of particle size and homogenization of size distribution were achieved, leading to a significant improvement in physicochemical stability. The obtained PTX-PTE NPs were characterized by PXRD, DSC, FT-IR, and SEM, and compared with PTX, PTE and the physical mixture of PTX + PTE. Ultimately, it was demonstrated that PTX-PTE NPs were amorphous spherical particles of nanoscale size formed by the non-covalent binding of PTX and PTE.

In vitro dissolution studies revealed that the dissolution capacity of this nanoscale supramolecular complex was markedly enhanced compared to the raw drug substance. This enhancement can be primarily attributed to the supramolecular interactions between PTX and PTE, which not only modify the crystalline structure of the drug but also improve its solubility in aqueous environments. However, the use of SDS solution as the dissolution medium for PTX-PTE NPs is only applicable for preliminary screening under idealized conditions to validate the optimization of the poorly soluble drug PTX through nano and co-amorphous strategies. This choice is justified by the need for a discriminating and robust medium that can achieve sink conditions for highly hydrophobic drugs like PTX, thereby preventing dissolution from being the rate-limiting step in assessing the performance of the novel formulations [52,53,54]. Compared with similar literature, in a dissolution experiment of the co-amorphous form of PTX and curcumin, the solubility of the co-amorphous form of PTX and curcumin was significantly increased compared to the raw material of paclitaxel, rising from 0.46 μg/mL to 1.6 μg/mL in pH 1.2 HCl solution (*p* < 0.01). This indicates that amorphizing drugs is an effective way to improve the water solubility of drugs [55].

An in vitro pharmacodynamic evaluation and investigation into the mechanism of combination therapy were conducted on PTX-PTE NPs. CCK-8 assay results indicated that the complex exerted a stronger inhibitory effect on A549/T cells than the individual drug components. Further mechanistic studies revealed that the combination therapy effectively suppressed the expression of P-gp and CDK1 proteins in A549/T cells, thereby significantly enhancing the anti-A549/T activity of PTX. These findings suggest that incorporating PTE may improve the anti-A549/T efficacy of PTX, potentially by reversing multidrug resistance (MDR) through modulation of drug resistance-related protein expression [56].

By constructing a drug supramolecular complex composed of PTX and PTE, this study successfully improved drug solubility, thereby enhancing in vitro pharmacodynamic outcomes. The development of a single supramolecular complex containing two pharmacologically active drugs provides a novel strategy for combination therapy. This approach holds potential to mitigate unfavorable physicochemical properties, simplify administration protocols, optimize dosing regimens, and ultimately improve patient compliance. However, these potential advantages still require validation through selected in vitro studies under more physiologically relevant conditions in future research, including drug release investigations, Caco-2 cell monolayer transport models, and 3D tumor cell models to obtain experimental data with enhanced in vivo relevance.

## 4. Materials and Methods

### 4.1. Materials

PTX (MW: 853.9) ≥99.5% (Jinhe Biopharmaceutical Co., Ltd., Shanghai, China), PTE (MW: 256.3) 98% (Zesheng Technology Co., Ltd., Anhui, China), anhydrous ethanol ≥99% (Kolon Chemicals Co., Ltd., Chengdu, China), PVP-K30 (Meril Chemical Technology Co., Ltd., Shanghai, China), Enhanced Cell Counting Kit-8 (C0042, Beyotime Biotechnology Co., Ltd., Shanghai, China), P-glycoprotein antibody (P-gp) and Cyclin-dependent kinase 1 antibody (CDK1) (Abgent, Wuhan, China), fetal bovine serum (Tianhang Biotechnology Co., Ltd., Huzhou, China), Ham’s F-12K cell culture medium (Procell Life Science & Technology Co., Ltd., Wuhan, China), Dialysis bag (regenerated cellulose membrane, MWCO: 3500 Da; Yi Bo Biotechnology Co., Ltd., Changsha, China), RIPA lysis buffer (P0013B, Beyotime Biotechnology Co., Ltd., Shanghai, China), BCA protein assay kit (P0010, Beyotime Biotechnology Co., Ltd., Shanghai, China), SDS-PAGE loading buffer (P0015, Beyotime Biotechnology Co., Ltd., Shanghai, China), SDS-PAGE pre-cast gels (P0056A, Beyotime Biotechnology Co., Ltd., Shanghai, China), ECL chemiluminescence kit (P0018AS, Beyotime Biotechnology Co., Ltd., Shanghai, China), horseradish peroxidase (HRP)-labeled goat anti-mouse IgG(H + L) (A0216, Beyotime Biotechnology Co., Ltd., Shanghai, China). The A549 cells and A549/T cells were provided by Guangxi Key Laboratory of Efficacy Study on Chinese Materia Medica of Guangxi University of Chinese Medicine, Nanning, Guangxi, China. HPLC analysis experiments use deionized water, while all other experiments use ultrapure water (Milli-Q).

### 4.2. Preparation of PTX-PTE NPs

The preparation of PTX-PTE NPs via the antisolvent precipitation method involved systematic control of variables, including a screening study of PTX-to-PTE molar ratios (2:1, 1:1, 1:2, 1:3, 1:4), PVP-K30 dosage (mg) (0.5, 1.5, 2.5, 3.5), organic-to-aqueous phase ratio screening (1:6, 1:8, 1:10, 1:12), reaction temperature control (30 °C, 35 °C, 40 °C), and PTX dosage (mg) (10, 15, 20, 25). Using particle size and PDI as optimization indicators, single-factor investigations and orthogonal experimental designs were conducted to determine the optimal preparation process. All subsequent characterization samples were fabricated according to the finalized formulation and process.

Optimal prescription process: dissolve 30 mg of PVP-K30 in 20 mL of ultrapure water and place it on a magnetic stirrer to stir and heat it as the aqueous phase; dissolve 30 mg of PTX (853.91 g/mol) and 26.9 mg of PTE (256.28 g/mol) together in 2 mL of ethanol as the organic phase. Heat both the aqueous and organic phases to 35 °C, then dropwise add the organic phase to the aqueous phase, continue stirring for 5 min, and obtain a white suspension. Dialyze the suspension with a dialysis bag for 5 h, and the PTX-PTE NPs are obtained.

### 4.3. Preparation of Physical Mixed Samples (PTX + PTE)

PTX + PTE were prepared by combining the two compounds in a 1:3 molar ratio and thoroughly mixing them using a mortar and pestle.

### 4.4. Particle Size Analysis

Take 0.1 mL of PTX-PTE NPs and place it in 0.9 mL of ultrapure water. Then, conduct particle size analysis using the Mastersizer 2000 (Malvern Panalytical Ltd., Malvern, UK). Repeat the sample three times.

### 4.5. Sample Pretreatment for PXRD, DSC and FT-IR

Transfer an appropriate volume of PTX-PTE NPs suspension into a centrifuge tube and centrifuge at 7500 rpm for 5 min. After centrifugation, carefully pour out the supernatant and retain the precipitate. Then, air-dry the precipitate at room temperature until the solvent is completely evaporated for subsequent experiments.

### 4.6. Powder X-Ray Diffraction (PXRD)

An appropriate amount of the processed sample was evenly spread onto the surface of the sample plate and gently pressed to ensure a flat surface with uniform thickness. For testing, a Rigaku MiniFlex 600 diffractometer (Rigaku, Tokyo, Japan) was employed under the following conditions: Cu Kα radiation source (wavelength = 1.5406 Å), voltage = 40 kV, current = 15 mA. Data for each sample group were recorded over a 2*θ* range of 3 to 40 degrees, with a scanning speed of 10 degrees per minute

### 4.7. Thermal Analysis

The NETZSCH STA 449 synchronous thermal analyzer (NETZSCH Company, Selb, Bavaria, Germany) was employed, with high-purity nitrogen serving as the protective gas. A precisely weighed 5 mg sample of dried PTX-PTE NPs was placed in an aluminum crucible. The analysis was conducted over a temperature range of 25 to 400 °C, with a controlled heating rate of 10 °C/min.

### 4.8. Fourier Transform Infrared Spectroscopy (FT-IR)

Take 2.5 mg of solid powder of the sample to be tested, add 200 mg of fine potassium bromide powder, mix evenly and then press into a tablet. Keep it under a pressure of 50 kN on the SSP-10A tablet press (Shimadzu International Trading Co., Ltd., Kyoto, Japan) for 3 min to obtain the sample that meets the requirements. The Fourier transform infrared spectrometer (Nicolet, Madison, WI, USA) was used under the conditions of a scanning range of 4000–400 cm^−1^, a spectral resolution of 2 cm^−1^, and an average of 64 scans. The results were visualized in the Origin analysis software (OriginPro 9.0 64 Bit, OriginLab, Northampton, MA, USA).

### 4.9. Scanning Electron Microscope (SEM) Analysis

Tests were conducted using a Quanta 250 scanning electron microscope (FEI, Hillsboro, OR, USA). Take an appropriate amount of PTX, PTE, PTX + PTE and PTX-PTE NPs, and fix them on the sample stage with double-sided tape. Then, perform gold-palladium sputtering coating on the samples under vacuum conditions and place them on the sample stage for observation. The raw drug samples and PTX-PTE NPs solid samples are observed at a magnification of 10,000 times.

### 4.10. In Vitro Dissolution Experiment

The dissolution experiment was performed in a 20 mL medium consisting of deionized water with 0.5% Sodium dodecyl sulfate (SDS), under conditions of 100 rpm stirring and a temperature of 37 °C. At predetermined time intervals, approximately 3 mL of the solution was sampled from the container and replenished with an equivalent volume of fresh dissolution medium. Following filtration, the concentrations of PTX (measured at 227 nm) and PTE (measured at 206 nm) were quantified using an Agilent 1260 II high-performance liquid chromatograph (Agilent Technologies, Santa Clara, CA, USA).

### 4.11. In Vitro Cell Cytotoxicity Tests

In vitro cytotoxicity assays were performed using the human lung adenocarcinoma cell line A549 and its paclitaxel-resistant variant, A549/T. A549/T cells were seeded into 96-well plates containing Ham’s F-12K medium supplemented with 10% fetal bovine serum, 0.5 μg/mL PTX, and 1% penicillin-streptomycin solution, whereas the culture medium for A549 cells lacked PTX. The cells were maintained in a humidified incubator at 37 °C under a 5% CO_2_ atmosphere. Following cell attachment, the medium was removed, and the cells were washed with phosphate-buffered saline (PBS). Subsequently, a dosing volume of 0.1 mL per well in 96-well plates was administered, with culture medium used as the diluent and DMSO content controlled as a solubilizer not exceeding 0.1%. For A549 cells, the concentrations of PTX (ng/mL) in the PTX group, PTX + PTE group, and PTX-PTE NPs group were 0, 15.6, 31.2, 62.5, 125, 250, and 500, while the concentration of PTE (μg/mL) in the PTE group was 0, 0.39, 0.78, 1.56, 3.125, 6.25, 12.5, and 2.5. The cells were incubated at 37 °C, 5% CO_2_, and 95% relative humidity for 24 h. For A549/T cells, the concentrations of PTX (μg/mL) in the PTX group, PTX + PTE group, PTX-PTE NPs group, and PTE group were 0, 0.78, 1.56, 3.125, 6.25, 12.5, and 25. In the PTE group of A549/T, the dosing concentration was calculated based on a PTX:PTE (1:3) molar ratio to determine the mass concentration. After incubation, observations were made at 24 h, 48 h, and 72 h. After treatment, the culture medium was replaced with CCK-8 solution, and 0.1 mL was added to each well. The plates were further incubated at 37 °C for 3 h. Finally, absorbance was measured at 450 nm using a multi-functional microplate reader, with each concentration tested in triplicate. Cell viability was then calculated using the following formula:

$$Cell\;viability\left(\%\right)=\frac{A_{experimental}-A_{blank}}{A_{control}-A_{blank}}\times 100\%$$

### 4.12. Western Blotting

After lysis with pre-cooled RIPA buffer, protein extracts from A549/T cells of the Blank group, PTX group, PTE group, and PTX-PTE NPs group were obtained by centrifugation and supernatant collection. Protein concentration was quantified using the BCA method and adjusted to uniform levels. Separation was performed via SDS-PAGE pre-cast gels (electrophoresis buffer: 25 mM Tris/250 mM Glycine/0.1% SDS) under constant voltage (80 V, 60 min), followed by semi-dry transfer (transfer buffer: 25 mM Tris/192 mM Glycine/20% methanol) onto a nitrocellulose membrane (200 mA, 30 min). The membrane was blocked with 5% skim milk-PBS for 1 h at room temperature, incubated sequentially with specifically diluted primary antibody (overnight at 4 °C) and HRP-conjugated secondary antibody (2 h at room temperature), washed three times with PBST buffer solution (NaCl: 137 mM, KCl: 2.7 mM, Na_2_HPO_4_: 10 mM, KH_2_PO_4_: 1.8 mM, 0.1% Tween-20), and finally visualized using ECL chemiluminescent substrate. Images were captured by a Tanon 4600 imaging system (Tanon Science & Technology Co., Ltd., Shanghai, China) and quantitatively analyzed with ImageJ 1.8.0 software.

### 4.13. Statistical Analysis

Statistical analysis was performed using GraphPad Prism 9.5. Data are presented as mean ± SD. Comparisons between two groups were made using an unpaired t-test, while comparisons among multiple groups were analyzed by one-way ANOVA followed by Tukey’s post hoc test. A P value of less than 0.05 was considered statistically significant (* *p* < 0.05, ** *p* < 0.01, *** *p* < 0.001, **** *p* < 0.0001).

## 5. Conclusions

This study aims to advance the synergistic application of PTX through co-amorphous systems and multi-drug combinatorial strategies, thereby enhancing therapeutic efficacy. PTX-PTE NPs were successfully prepared by optimizing the anti-solvent precipitation method, with an average particle size of approximately 150 nm and a PDI below 0.3, demonstrating excellent size uniformity. Characterization results confirmed that the complex has an amorphous nanostructure, and the phenolic hydroxyl groups of PTE form stable bonds with the carbonyl groups of PTX through hydrogen bonding. This nanoformulation increased the dissolution rate of paclitaxel, with the peak concentration reaching seven times that of the raw material drug. In vitro cytotoxicity experiments showed that PTX-PTE NPs exhibited enhanced cytotoxicity against both A549 and its drug-resistant strain A549/T cells, which may be related to the downregulation of P-gp and CDK1 expression, demonstrating the potential of PTX-PTE NPs in addressing PTX-resistant tumor cells. The results of this study demonstrate the strong clinical potential of the co-amorphous strategy, which enables multi-component co-delivery and enhances drug solubility through nanoscale features. Further development of this technology may help overcome key challenges in conventional formulations and advance more precise drug delivery systems.

## Figures and Tables

**Table 1:** The influence of the molar ratio of PTX to PTE in P PTX-PTE NPs on particle size and PDI.

The Molar Ratio of PTX to PTE	Particle Size (nm)	PDI
2:1	179.50 ± 2.0	0.19 ± 0.012
1:1	163.00 ± 1.6	0.08 ± 0.012
1:2	158.40 ± 0.1	0.19 ± 0.012
1:3	145.87 ± 1.4	0.17 ± 0.016
1:4	174.87 ± 1.1	0.06 ± 0.011

**Table 2:** The influence of the dosage of PVP-K30 in PTX-PTE NPs on particle size and PDI.

The Dosage of PVP-K30 (mg/mL)	Particle Size (nm)	PDI
0.5	185.6 ± 2.5	0.258 ± 0.004
1.5	143.1 ± 1.2	0.170 ± 0.014
2.5	182.0 ± 0.3	0.091 ± 0.004
3.5	382.2 ± 9.3	0.327 ± 0.009

**Table 3:** The influence of the ratio of organic phase to aqueous phase in PTX-PTE NPs on particle size and PDI.

The Ratio of Organic Phase to Aqueous Phase	Particle Size (nm)	PDI
1:6	161.0 ± 1.0	0.068 ± 0.011
1:8	183.0 ± 1.5	0.067 ± 0.016
1:10	143.1 ± 1.2	0.170 ± 0.014
1:12	188.3 ± 2.7	0.060 ± 0.000

## Data Availability

The original contributions presented in this study are included in the article/Supplementary Material. Further inquiries can be directed to the corresponding author.

## Supplementary Materials

The following supporting information can be downloaded at: https://www.mdpi.com/article/10.3390/ph18121828/s1. Table S1. The influence of paclitaxel dosage in PTX-PTE NPs on particle size and polydispersity index, Table S2. The influence of temperature in PTX-PTE NPs on particle size and polydispersity index, Table S3. Test factors and levels, Table S4. Orthogonal test scheme and Figure S1. The effects of different PTE concentrations and culture times on A549 cells.

## Abbreviations

The following abbreviations are used in this manuscript:

PTX	Paclitaxel
PTE	Pterostilbene
PTX + PTE	The physical mixture of PTX and PTE
PTX-PTE NPs	paclitaxel-pterostilbene nanoparticles
SDS	Sodium dodecyl sulfate
DSC	Differential Scanning Calorimetry
PXRD	Powder X-ray diffraction
FT-IR	Fourier transform infrared spectroscopy
SEM	Scanning electron microscope
CDK1	Cyclin-Dependent Kinase 1
P-gp	P-glycoprotein
