# Supplementary material for: Enhanced Anti-Tumor Efficacy of Paclitaxel Nanoparticles via Supramolecular Self-Assembly with Pterostilbene
Source: Pharmaceuticals (Basel). 2025 Dec 1;18(12):1828. doi: 10.3390/ph18121828 (PMC12735992; doi:10.3390/ph18121828)
Supplement: Supplementary file 1 [file pharmaceuticals-18-01828-s001.zip › pharmaceuticals-3909139-supplementary.pdf]

Supplementary Information

Table S1. The influence of paclitaxel dosage in PTX–PTE NPs on particle size and polydispersity index.

The dosage of PTX (mg/mL)	Particle size (nm)	PDI
10	109.6±0.300	0.054±0.012
15	137.6±3.001	0.142±0.014
20	143.1±1.217	0.170±0.014
25	161.2±0.625	0.084±0.008

Table S2. The influence of temperature in PTX–PTE NPs on particle size and polydispersity index.

Temperature (℃)	Particle size (nm)	PDI
30	＞1000	＞0.300
35	137.6±3.001	0.142±0.014
40	145.1±1.594	0.05±0.026

Table S3. Test factors and levels

Level	(A) PVP–K30 (mg/mL)	(B) The ratio of organic phase to water	(C) PTX (mg)
1	0.5	1:8	15
2	1.5	1:10	20
3	2.5	1:12	25

Table S4. Orthogonal test scheme

Level	(A) PVP–K30 (mg/mL)	(B) The ratio of organic phase to water	(C) PTX (mg)	Particle size (nm)
1	1	1	1	175.1
2	1	2	3	198.5
3	1	3	2	157.3
4	2	1	3	150
5	2	2	2	153.1
6	2	3	1	137.1
7	3	1	2	745
8	3	2	1	294.3
9	3	3	3	379.9
K1	530.9	1070.1	606.5	
K2	440.2	645.9	1055.4	
K3	1419.2	674.3	728.4	
k1	176.97	356.7	202.17	
k2	146.73	215.3	351.8	
k3	473.07	227.77	242.8	
R	326.34	141.4	149.63	
the best level		$A_2B_2C_1$		

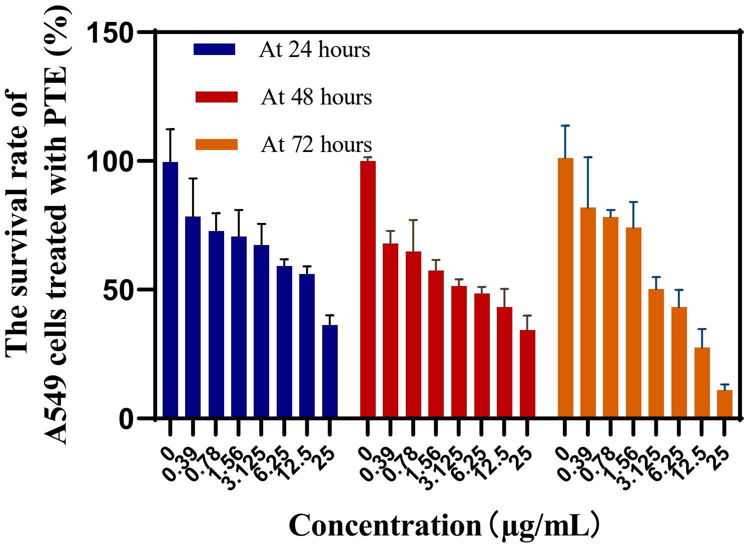

Fig. S1. The effects of different PTE concentrations and culture times on A549 cells.
